# Epidemiology, Clinico-Pathological Characteristics, and Comorbidities of SARS-CoV-2-Infected Pakistani Patients

**DOI:** 10.3389/fcimb.2022.800511

**Published:** 2022-05-26

**Authors:** Saadia Omer, Mehrunnisa Fatima Gondal, Muhammad Usman, Muhammad Bilal Sarwar, Muhammad Roman, Alam Khan, Nadeem Afzal, Tanveer Ahmed Qaiser, Muhammad Yasir, Faheem Shahzad, Romeeza Tahir, Saima Ayub, Javed Akram, Raja Muhammad Faizan, Muhammad Asif Naveed, Shah Jahan

**Affiliations:** ^1^Department of Immunology, University of Health Sciences, Lahore, Pakistan; ^2^Institute of Public Health, Health Department, Government of Punjab, Lahore, Pakistan; ^3^Department of Community Medicine, Fatima Jinnah Medical University, Lahore, Pakistan; ^4^Allama Iqbal Medical College, Jinnah Hospital, Lahore, Pakistan; ^5^Department of Molecular Biology, Shaheed Zulfiqar Ali Bhutto Medical University, Islamabad, Pakistan; ^6^Quadram Institute Bioscience, Norwich Research Park, Norwich, United Kingdom; ^7^Department of Medicine, Shifa College of Medicine, Islamabad, Pakistan

**Keywords:** SARS-CoV-2, COVID-19, Acute Respiratory Distress Syndrome (ARDS), clinic-pathological characteristics, comorbidities, medication, ICU vs. non-ICU, Lungs-CT-scan

## Abstract

SARS-CoV-2 is a causative agent for COVID-19 disease, initially reported from Wuhan, China. The infected patients experienced mild to severe symptoms, resulting in several fatalities due to a weak understanding of its pathogenesis, which is the same even to date. This cross-sectional study has been designed on 452 symptomatic mild-to-moderate and severe/critical patients to understand the epidemiology and clinical characteristics of COVID-19 patients with their comorbidities and response to treatment. The mean age of the studied patients was 58 ± 14.42 years, and the overall male to female ratio was 61.7 to 38.2%, respectively. In total, 27.3% of the patients had a history of exposure, and 11.9% had a travel history, while for 60% of patients, the source of infection was unknown. The most prevalent signs and symptoms in ICU patients were dry cough, myalgia, shortness of breath, gastrointestinal discomfort, and abnormal chest X-ray (*p* < 0.001), along with a high percentage of hypertension (*p* = 0.007) and chronic obstructive pulmonary disease (*p* = 0.029) as leading comorbidities. The complete blood count indicators were significantly disturbed in severe patients, while the coagulation profile and D-dimer values were significantly higher in mild-to-moderate (non-ICU) patients (*p* < 0.001). The serum creatinine (1.22 μmol L^-1^; *p* = 0.016) and lactate dehydrogenase (619 μmol L^-1^; *p* < 0.001) indicators were significantly high in non-ICU patients, while raised values of total bilirubin (0.91 μmol L^-1^; *p* = 0.054), C-reactive protein (84.68 mg L^-1^; *p* = 0.001), and ferritin (996.81 mg L^-1^; *p* < 0.001) were found in ICU patients. The drug dexamethasone was the leading prescribed and administrated medicine to COVID-19 patients, followed by remdesivir, meropenem, heparin, and tocilizumab, respectively. A characteristic pattern of ground glass opacities, consolidation, and interlobular septal thickening was prominent in severely infected patients. These findings could be used for future research, control, and prevention of SARS-CoV-2-infected patients.

## Introduction

The novel coronavirus (2019-nCoV) was first identified in patients with pneumonia of unknown cause, originating in Wuhan, China, in late December 2019 (Lu et al., 2020; Zhou et al., 2020). The virus that caused this infection belongs to the Conronaviridae family (Lvov and Alkhovsky, 2020), which was later named “severe acute respiratory syndrome coronavirus 2” (SARS-CoV-2) by the World Health Organization (WHO) and caused coronavirus disease 2019 (COVID-19) (WHO, 2020). In January 2020, WHO declared the COVID-19 outbreak, a public health emergency of international concern, and a pandemic in March 2020 (Sun et al., 2020a). There have been around half a billion confirmed cases, with approximately 5 million deaths attributed to the ongoing COVID-19 pandemic, while the confirmed cases in Southeast Asia are in the range of 50 million (WHO, 2021; WHO, 2022).

In Pakistan, the first confirmed case of COVID-19 was reported on February 26, 2020, and that spread horrifically in the following months in all parts of the country despite the safety measures adopted (Khalid and Ali, 2020; Mushtaq et al., 2020). Initially, due to the lack of access to essential healthcare services at the Pak-Iran border, the asymptomatic pilgrims returning from Iran to Pakistan introduced the virus in the latter (Javed et al., 2021). Later, over the period, unchecked non-essential international travel to Pakistan has led to its spread in all parts of the country (Nawaz et al., 2020). Considering the severity of the pandemic, the government of Pakistan has established the National Command and Operational Centre (NCOC) to synergize and articulate national efforts against COVID-19 (NCOC, 2021a). The measure taken by NCOC to control the pandemic was quite effective. NCOC adopted the WHO checklist to exercise travel restrictions, placed smart lockdowns, enforced workplace hazard controls, and even implemented facility closures to minimize exposure with the carriers (asymptomatic) as preventive measures (NCOC, 2021b). Pakistan’s overall verified COVID-19 count has reached around one and a half million, with approximately 90% recoveries and 2% fatalities (NCOC 2022). Despite the control measures launched into action, the alarming rise in COVID-19 cases and the increasing fatalities in the region have raised multiple concerns regarding the infrastructure of our healthcare system and the overwhelming burden placed on it. Understanding the relationship between COVID-19 and epidemiological features like clinicopathological characteristics and treatment available for mild-to-moderate and severe patients is essential to suggest preventive measures. The epidemiological features include age, sex, race, and comorbidities, which are the most studied parameters not only in COVID-19 but also in other infectious diseases (Bi et al., 2020; Sun et al., 2020b, Yang et al., 2020).

Practically, most health workers relied upon clinical signs and symptoms, mainly of the respiratory and digestive system, associated fever, fatigue, and lab findings—such as values of IL-6, D-dimer, procalcitonin, complete blood count (CBC), *etc*.—to assess the disease prognosis (Grant et al., 2020; Lechien et al., 2020; Tian et al., 2020). The SARS-CoV-2-infected patients showed variable symptoms, ranging from mild to severe illness. It primarily affects the pulmonary system, causes symptoms like sore throat, cough, rhinorrhea, nasal congestion, and dyspnea; digestive system-related complaints causing nausea, vomiting, diarrhea, and abdominal pain; and associated systemic symptoms like fever, headache, myalgia, arthralgia, generalized body ache, and fatigue (Mehta et al., 2021). It may affect the nervous system, causing atypical clinical findings such as anosmia, loss of taste, dizziness, and, rarely, seizure (Niazkar et al., 2020; Vohora et al., 2020). The most common symptoms seen were fever, dyspnea, myalgia, *etc*. One in five patients infected with SARS-CoV-2 did not develop noticeable symptoms, thus acting as a silent carrier (He et al., 2021) and thereby increasing the chances of the further progression of SARS-CoV-2 infection in the community as well as putting at risk old-age patients and those with associated comorbidities like diabetes, hypertension, chronic obstructive pulmonary disease (COPD), acute respiratory distress syndrome (ARDS), ischemic heart disease (IHD), chronic liver disease, chronic kidney disease (He et al., 2020a). The comorbidities and the nature of pathological findings related to organs and tissues are the leading factors that determine the disease severity and outcome (Gu and Korteweg, 2007; Mueller et al., 2020).

The mortality in COVID-19 depends on the virus–host genetic interaction and geographical setting (international and regional spread), as climate differences influence viral transmission *via* respiratory droplets (Oberemok et al., 2020). Punjab is the largest and most densely populated province of Pakistan and has a population of 110 million, out of which about 64% reside in urban areas (GOP, 2017). It has a high population density of about 536 persons per square kilometer, increasing the susceptibility towards the spread of COVID-19. Out of the total number of confirmed COVID-19 patients, about 37% were located in the province of Punjab (GOP, 2021). Islamabad is the tertiary capital of Pakistan with more than 2 million inhabitants, but due to its limited area, it has the highest average population density of 2,215 persons per square kilometer (GOP, 2017). The city is the gateway to the country for foreign travellers and provides habitation to people from the whole country. Accordingly, the main referral hospital of Islamabad was also selected for this study.

Only a few studies have been undertaken in Pakistan to determine the epidemiological aspect, clinic pathological characteristics, and comorbidities of COVID-19 patients. A key shortcoming of several COVID-19 studies seems to be the focus on either epidemiological or clinico-pathological factors. This study aims to give insight into the interrelationships between illness and epidemiology, clinicopathological characteristics, co-morbidities, and treatment options for moderately and critically ill patients. The goal of this study is to understand more about the COVID-19 pandemic by evaluating symptomatic hospitalized COVID-19 patients based on their epidemiological, clinical, and laboratory features. This comparative cross-sectional study was conducted in leading hospitals designated for COVID-19 treatments, ie., Mayo Hospital Lahore, Jinnah Hospital Lahore, Sheikh Zaid Hospital Lahore, University of Health Sciences Lahore, Nishtar Hospital Multan, Victoria Hospital Bahawalpur, Pakistan Institute of Medical Sciences Islamabad, and Infectious Treatment Centre Islamabad. The findings of this pioneering study will serve as a foundation for policy development and strategic planning to prevent, diagnose, and cure the COVID-19 pandemic.

## Materials and Methods

### Sampling Technique

A non-probability convenience sampling technique was used for the selection of the study population. Quantitative real-time polymerase chain reaction (qPCR) confirmed the SARS-CoV-2-infected patients, with age >18 years, who were admitted in the isolation wards of the selected hospitals and selected after taking permission from the respective administration and obtaining their informed consent. None of the patients in this study had previously been vaccinated or had a history of the illness. Furthermore, patients with co-infections were ruled out, with the help of a clinician, using available laboratory tests and other clinical data; however, patients with an asymptomatic illness in the past could not be ruled out entirely. Patients with co-infections of *Pseudomonas* spp., *Klebsiella pneumoniae*, *Streptococcus pneumoniae*, *Acinetobacter* spp., and *Escherichia coli* were identified and excluded from this study.

These patients were classified into mild-to-moderate and severe/critical categories using the operational definitions of the “Chinese Clinical Guidance for COVID-19 Pneumonia Diagnosis and Treatment”, published by the Chinese National Health Commission (Gao et al., 2020). The patients in the isolation wards, who meet the case definition for COVID-19 with and without evidence of pneumonia, were categorized in the mild-to-moderate group, whereas the patients in intensive care units who had clinical signs of pneumonia (fever, cough, dyspnea, and tachypnea)—with the following parameters: respiratory rate, >30 breaths min^-1^; severe respiratory distress; or oxygen saturation (SpO_2_) <90% on room air—were considered in the severe group. The estimated sample size, using the WHO formula and considering the anticipated population proportion, was ~450 patients (Yang et al., 2020).

### Data Collection/Measurements

Before entering the isolation wards, the guidelines and SOPs of each hospital were adopted. A detailed questionnaire was prepared with the assistance of three medical consultants for information collection, which was based on similar studies (Al Mutair et al., 2020; Kirchberger et al., 2021; Yegorov et al., 2021). It included demographic data (age and gender), family type (nuclear and extended), source of infection (travel history and contact with COVID-19 patient), etiology, clinical features (upper and lower respiratory tract and gastro-intestinal tract symptoms), co-morbidities (diabetes mellitus, hypertension, chronic liver disease, chronic obstructive pulmonary disease, ischemic heart disease, cancer, tuberculosis, etc.), laboratory values (complete blood count, biochemical parameters, coagulation profile, inflammatory biomarkers for organ function, and analysis of immunological responses), treatment given (oral/IV antibiotics, antiviral, steroids, I/V fluids, orogastric fluids, antimalarial, and any experimental drug), length of hospitalization, and time taken to get the negative result of qPCR for SARS-CoV-2 (1st qPCR negative report). The digital images of the chest CT scan of COVID-19 patients were also obtained from selected hospitals and shared with experienced radiologists for characteristic disease findings.

### Statistical Analysis

The data were entered, cleaned, and analyzed using Statistical Package for Social Sciences, V.23. Quantitative variables like age and laboratory parameters were presented as mean and standard deviation. In contrast, qualitative data like clinical features, comorbidities, and demography were presented in frequency and percentages. The relationship between COVID-19 with clinico-pathological parameters and comorbidities among mild-to-moderate and severe patients was assessed using chi-square test (*p* ≤ 0.05). The means were compared using Student’s *t*-test or ANOVA where applicable. Bar and pie diagrams were used to present categorical data where applicable.

## Results

### Demography

**Figure 1:**
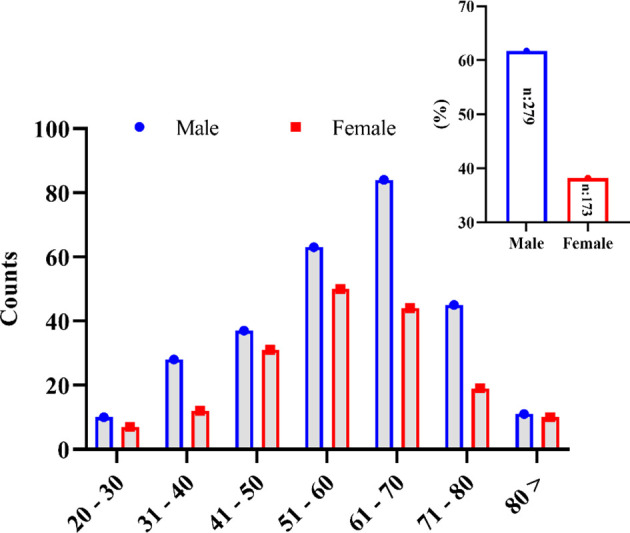
Gender-wise distribution of the SARS-CoV-2-infected patients in the different age groups studied in this study.

Four hundred fifty-two (452) patients confirmed to have COVID-19 diseases and admitted to five leading hospitals in different cities of the country were included in this study. Among these, 279 (61.7%) patients were male, and 173 (38.2%) were female (**Figure 1**). The mean age of the studied patients was 58 ± 14.42 years old. The overall patients’ age of <50 years was less in number (126, 27.88%; with a mean age of 40 ± 7.83 years) compared with patients aged ≥50 years (326, 72.12%; with a mean age of 65 ± 9.13 years). The mild-to-moderate (non-ICU) cases were higher with patients aged <50 years (83, 34.73%), whereas only 43 (20.19%) were severe (ICU) cases in this age group. In a group with patients >50 years old, more severe cases were seen, *i*.*e*., 170 (79.81%) compared with the 156 moderate cases (65.27%). Most of the patients belonged to the extended family type (294, 65%) compared with those who came from a nuclear family (158, 35%). However, the severity of the disease was insignificant between the extended and the nuclear family types (*p* = 0.429). Overall, 127 (27.3%) patients had a known history of exposure to the infected environment; 54 (11.9%) patients were with a travel history, while for 274 (60.6%) patients the cause of acquiring the infection was unknown. The severity of the disease was insignificant between the sources of COVID-19 infection (*p* = 0.496). The detailed demographic information is summarized in **Table 1**.

**Table 1:** Epidemiological data of 452 symptomatic COVID-19 patients.

Variables	Total (*n* = 452)	Mild/moderate (*n* = 239)	Severe (*n* = 213)	*p*-value
**Age (years)**	58.52 (14.42)	55.75 (15.1)	61.62 (13.0)	N/A
≤50 years, *n* (%) Mean (SD)	126 (27.88) 40.22 (7.83)	83 (34.73) 38.90 (8.18)	43 (20.19) 42.77 (6.57)	
>50 years, *n* (%) Mean (SD)	326 (72.12) 65.59 (9.13)	156 (65.27) 64.71 (8.81)	170 (79.81%) 66.39 (9.34%)	
**Gender**				
Male	279 (61.7%)	137 (49.2%)	142 (50.8%)	N/A
Female	173 (38.2%)	102 (58.9%)	71 (41.1%)	
**Family type**				
Nuclear	158 (35%)	88 (55.7%)	70 (44.3%)	0.429
Extended	294 (65%)	151 (51.4%)	143 (48.6%)	
**Source of COVID-19 infection**				
Travel history	55 (12.2%)	33 (60%)	22 (40%)	0.496
Contact history	124 (27.4%)	66 (53.2%)	58 (46.8%)	
No known source for COVID-19	273 (60.4%)	140 (51.3%)	133 (48.7%)	

Data are presented as n (%) or mean (SD) unless otherwise indicated. The P-values suggest the disparity between age groups, family type, and source of infection among mild/moderate and severe COVID-19 patients. The significance level for p value is ≤0.05.

### Clinical Features of SARS-CoV-2-Infected Patients

The most common symptom of illness in the COVID-19 patients was fever (*n* = 364, 80%), followed by dyspnea at rest (*n* = 343, 75%) and cough (*n* = 261, 57%). In a small percentage of COVID-19 patients, fatigue (*n* = 138, 30%), pneumonia (*n* = 89, 19%), myalgia and generalized body aches (*n* = 75, 16%), vomiting (*n* = 61, 13%), headache (*n* = 36, 7.9%), sore throat (*n* = 33, 7.3%), diarrhea (*n* = 33, 7.3%), sputum production (*n* = 19, 4.2%), nausea (*n* = 13, 2.9%), loss of taste (*n* = 12, 2.7%), rhinorrhea (*n* = 12, 2.7%), anosmia (*n* = 7, 1.5%), nasal congestion (*n* = 5, 1.1%), and abnormal chest X-ray (*n* = 36, 7.9%) were also observed. Some clinical manifestations differed significantly between mild-to-moderate and severe patients. The symptoms of dyspnea, pneumonia, and respiratory distress and abnormal chest X-ray findings were more pronounced in severe patients compared with the mild-to-moderate cases (*p* < 0.001). On the contrary, symptoms of fatigue (*p* < 0.001) and loss of taste (*p* = 0.04) were seen in the mild-to-moderate cases. A tabulated summary of the clinical features of SARS-CoV-2-infected patients is shown in **Table 2**.

**Table 2:** Clinical features of 452 symptomatic COVID-19 patients.

Clinical features	Total (*n* = 452)	Mild/moderate (*n* = 239)	Severe (*n* = 213)	*p*-value
Fever or chills	364 (80.5)	187 (78.2)	177 (83.1)	0.234
Shortness/difficulty of breathing	343 (75.9)	162 (67.8)	181 (84.9)	**0.000**
Cough	261 (57.7)	134 (56.1)	127 (59.6)	0.448
Fatigue	138 (30.5)	93 (38.9)	45 (21.1)	**0.000**
Pneumonia	89 (19.6)	16 (6.7)	73 (34.3)	**0.000**
Muscle or body aches	75 (16.6)	35 (14.6)	40 (18.8)	0.256
Vomiting	61 (13.5)	34 (14.2)	27 (12.7)	0.680
Headache	36 (7.9)	19 (7.9)	17 (7.9)	1.000
Abnormal chest X-ray	36 (7.9)	5 (2.1)	31 (14.6)	**0.000**
Sore throat	33 (7.3)	14 (5.9)	19 (8.9)	0.277
Diarrhea	33 (7.3)	15 (6.3)	18 (8.5)	0.469
Sputum production	19 (4.2)	13 (5.4)	6 (2.8)	0.240
Nausea	13 (2.9)	7 (2.9)	6 (2.8)	1.000
Loss of taste	12 (2.7)	10 (4.2)	2 (0.9)	**0.040**
Runny nose	12 (2.7)	9 (3.8)	3 (1.4)	0.149
Loss of smell	7 (1.5)	5 (2.1)	2 (0.9)	0.455
Congestion	5 (1.1)	2 (0.8)	3 (1.4)	0.670

Data are presented as n (%). The P-values suggest the disparity between mild/moderate and severe COVID-19 patients. The significance level for p-value is ≤0.050. Bold values indicates the significant difference at mentioned p value.

### COVID-19 Patients With Comorbidities

Compared with the mild-to-moderate patients, the severe patients had underlying co-morbidities, such as hypertension (*n* = 104, 48.8%), type 2 diabetes (*n* = 82, 38.5%), and ischemic heart disease (*n* = 36, 16.9%). Other diseases included chronic kidney disease (*n* = 10, 4.7%), asthma (*n* = 6, 2.8%), immunocompromised state (*n* = 6, 2.8%), COPD (*n* = 7, 3%), liver disease (*n* = 2, 0.9%), and history of smoking (*n* = 3, 1.4%). The severe patients included a significantly high percentage of hypertension (*p* = 0.007) and COPD (*p* = 0.029) cases. These co-morbidities impact the outcome of disease severity and COVID-19 mortality. A comprehensive comparison is shown in **Table 3**.

**Table 3:** Comorbidities of 452 symptomatic COVID-19 patients.

Co-morbidities	Total (*n* = 452)	Mild/moderate (*n* = 239)	Severe (*n* = 213)	*P*-value
Hypertension	190 (42.0)	86 (35.9)	104 (48.8)	0.007
Type 2 diabetes mellitus	157 (34.7)	75 (31.4)	82 (38.5)	0.115
Ischemic heart disease	66 (14.6)	30 (12.5)	36 (16.9)	0.230
Chronic kidney disease	19 (4.2)	9 (3.7)	10 (4.7)	0.646
Asthma	11 (2.4)	5 (2.1)	6 (2.8)	0.763
Immunocompromised state	10 (2.21)	4 (1.6)	6 (2.8)	0.527
Chronic obstructive pulmonary disease	8 (1.8)	1 (0.4)	7 (3.3)	0.029
Smoking	5 (1.1)	2 (0.8)	3 (1.4)	0.670
Liver disease	3 (0.7)	1 (0.4)	2 (0.9)	0.604

Data are presented as n (%). The P-values suggest the disparity between mild/moderate and severe COVID-19 patients. The significance level for p-value is ≤0.050.

### Laboratory Parameters

The mean values of CBC were significantly high in severe patients, compared with mild-to-moderate cases, in terms of WBC (14,864.70 ± 5,821.73 *vs*. 11,055.94 ± 4,344.7, *p* < 0.001), platelets (249,672 ± 104,826.59 *vs*. 246,447.36 ± 83,141.91, *p* = 0.004), and neutrophil (9.99 ± 4.93 *vs*. 8.19 ± 3.27, *p* < 0.001). Similarly, the coagulation profile (d-dimer) was significantly high in mild-to-moderate patients (2,610.00 ± 3,607.04) compared with severe patients [2,213.67 ± 2,823.36 (mg L^-1^), *p* < 0.001]. Regarding the liver function test, total bilirubin was significantly high in severe patients (0.91 ± 1.41), while it was 0.54 ± 0.25 in mild-to-moderate patients (μmol L^-1^; *p* = 0.054). For renal function test, serum creatinine was significantly high in the mild-to-moderate group (1.22 ± 0.64), while it was 0.81 ± 0.25 mol L^-1^; *p* = 0.016) in severe patients. Lactate dehydrogenase (LDH) was significantly high in the mild-to-moderate group (619.00 ± 257.32) than in the severe group (587.58 ± 178.31, *p* < 0.001). Furthermore, severe patients had significantly high values of C-reactive protein (CRP) and ferritin (84.68 ± 57.25 and 996.81 ± 892.21 mcg/liter; *p* < 0.001), respectively, compared with the mild-to-moderate group of SARS-CoV-2-infected patients. From illness to discharge, the average length of hospital stay was longer in severe patients (10.79 ± 7.00 compared with the mild-to-moderate patients at 5.89 ± 3.41; *p* < 0.001). The detailed comparison of laboratory parameters is presented in **Table 4**. Furthermore, the laboratory parameters were also analyzed based on comorbidities in COVID-19 patients, and none of the above-reported laboratory parameters were significantly different in patients with and without comorbidities. However, Hb and ALT were significantly high in COVID-19 patients with hypertension (HTN). Similarly, PT was significantly high in COVID-19 patients with type 2 diabetes mellitus, and the urea-N/urea ratio was high in patients with IHD. It is worth mentioning that we have only analyzed the data of comorbidities with a significant number of patients *i*.*e*., HTN (42%), T2DM (34%), and IHD (14%). The rest of the comorbidities were not reported significantly in the study population to be included in the analysis (immunocompromised state, 2.2%), liver disease (0.7%), COPD (1.8), asthma (2.4%), and smoking (1.1%; **Supplementary Data).**

**Table 4:** Laboratory features of 452 symptomatic COVID-19 patients.

Laboratory parameters		Mild/moderate	Severe	*p*-value
**Complete blood count**				
Hb, g/dl	12.64 (2.15)		12.67 (2.27)	0.966
WBC/cubic, mm	11,055.94 (4,344.73)		14,864.70 (5,821.73)	0.000
Hematocrit, %	36.19 (6.87)		38.46 (6.67)	0.431
Platelets/cm	246,447.36 (83,141.91)		249,672.91 (104,826.59)	0.004
Neutrophils	8.19 (3.27)		9.99 (4.93)	0.000
Lymphocytes	1.47 (0.83)		1.47 (1.18)	0.458
Monocytes	0.40 (0.35)		0.48 (0.64)	0.459
**Coagulation profile**				
D-dimer	2,610.00 (3,607.04)		2,213.67 (2,823.36)	0.000
INR	1.16 (0.17)		1.28 (0.52)	0.198
APTT/s	30.36 (6.16)		31.07 (7.21)	0.606
PT/s	13.68 (2.46)		15.34 (7.25)	0.206
**Electrolytes and renal profile**				
Creatinine	1.22 (0.64)		0.81 (0.25)	0.016
Sodium	146.93 (10.55)		140.32 (5.09)	0.779
Potassium	4.38 (0.49)		4.57 (0.51)	0.198
**Liver function test**				
ALT/SGPT (U/L)	55.39 (45.20)		48.60 (26.04)	0.388
Total bilirubin	0.54 (0.25)		0.91 (1.41)	0.054
Inflammatory markers				
CRP	82.85 (44.52)		84.68 (57.25)	0.001
LDH	619.00 (257.32)		587.58 (178.31)	0.000
Ferritin	597.87 (390.37)		996.81 (892.21)	0.000
Procalcitonin	0.21 (0.19)		2.79 (5.96)	0.093
ESR	30.67 (11.59)		48.67 (29.87)	0.681
Other				
Troponin	11.27 (21.98)		78.38 (180.36)	0.154
Hospital duration (number of days)	5.89 (3.41)		10.79 (7.00)	0.000

Data are presented as mean (SD). The P-values suggest the disparity between mild/moderate and severe COVID-19 patients. The significance level for p-value is ≤0.050.

### Medications Used in the Treatment

In **Table 5**, shown is a summary of the medication given to the SARS-CoV-2-infected patients. Almost all SARS-CoV-2-infected patients, spanning between mild-to-moderate and severe, had a combination of antiviral, antibacterial, and corticosteroid-based medication combined with oxygen therapy. The patients were treated for bacterial infection from the following sources: *Pseudomonas* spp., *Klebsiella pneumoniae*, *Streptococcus pneumoniae*, *Acinetobacter* spp., and *Escherichia coli*. Only two critically ill patients were on invasive ventilators, while others were managed with non-invasive ventilation. Compared with the mild-to-moderate patients, a significant percentage of severe patients were treated with dexamethasone (*n* = 211, 99 *vs*. 207; 86.6%, *p* < 0.001), remdesivir (*n* = 178; 83.5 *vs*. 136; 56.9%, *p* < 0.001), meropenem (*n* = 121; 56.8% *vs*. *n* = 76; 31.8%, *p* < 0.001), heparin (*n* = 112; 52.6% *vs*. *n* = 75; 31.4%; *p <*0.001), and tocilizumab (*n* = 14; 6.6% *vs*. *n* = 1; 0.42%; *p* < 0.001).

**Table 5:** Treatment of 452 symptomatic COVID-19 patients.

Treatment	Total (*n* = 452)	Mild/moderate (*n* = 239)	Severe (*n* = 213)	*P*-value
Dexamethasone	418 (92.5)	207 (86.6)	211 (99)	**0.000**
Remdesivir	314 (69.4)	136 (56.9)	178 (83.5)	**0.000**
Azithromycin	206 (45.6)	119 (49.8)	87 (40.8)	0.059
Meropenem	197 (43.6)	76 (31.8)	121 (56.8)	**0.000**
Clexane	192 (42.5)	100 (41.8)	92 (43.2)	0.776
Heparin	187 (41.4)	75 (31.4)	112 (52.6)	**0.000**
Fluoroquinolones	152 (33.6)	87 (36.4)	65 (30.5)	0.196
Tocilizumab	15 (3.3)	1 (0.42)	14 (6.6)	**0.000**

Data are presented as n (%). The P-values suggest the disparity between mild/moderate and severe COVID-19 patients. The significance level for p-value is ≤0.050.

Bold values indicates the significant difference at mentioned p value.

### Correlation Between Laboratory Parameters

A significant correlation was assessed among laboratory parameters (the details of which are summarized in **Table 6**), namely:

- A highly significant positive correlation was evaluated between ferritin and Hb, WBC, ALT/SGPT, total bilirubin, CRP, and LDH, while it had a highly negative correlation with lymphocytes.
- D-dimer had a highly significant positive correlation with WBC, INR, procalcitonin, and LDH.
- Hb had a highly significant positive correlation with ALT/SGPT and hematocrit. In contrast, it was negatively correlated with CRP and sodium.
- WBC had a highly significant positive correlation with neutrophil, LDH, CRP, and INR.
- A highly significant positive correlation was assessed between hematocrit and ALT/SGPT.
- INR had a highly significant positive correlation with neutrophils.
- CRP had a highly significant positive correlation with procalcitonin, neutrophil, and LDH, whereas it was negatively correlated with potassium.
- LDH had a highly positive correlation with neutrophils.
- The platelets had a negative correlation with sodium and total bilirubin.

**Table 6:** Principal component analysis of the laboratory parameters.

Laboratory variable	Lymphocytes	Neutrophils	Ferritin	D-dimer	Troponin	LDH	CRP	Procalcitonin	Potassium	Sodium	Total bilirubin	ALT/SGPT	INR	Platelets	Hematocrit	WBC	Hb
Lymphocytes	1.000																
Neutrophils	0.004	1.000															
Ferritin	-.176^**^	.332^**^	1.000														
D-dimer	-0.056	.295^**^	.295^**^	1.000													
Troponin	.372^*^	0.099	0.181	0.074	1.000												
LDH	-0.026	.274^**^	.361^**^	.402^**^	-0.162	1.000											
CRP	-0.086	.216^**^	.302^**^	.143^*^	0.048	.267^**^	1.000										
Procalcitonin	-0.098	-0.020	0.250	.348^**^	0.155	-0.122	.415^**^	1.000									
Potassium	0.066	0.020	0.011	.133^*^	0.067	0.005	-.138^*^	-0.109	1.000								
Sodium	0.051	0.079	0.009	0.039	0.121	0.135	-0.078	-0.076	-0.021	1.000							
Total bilirubin	-0.079	.152^*^	.216^**^	.153^*^	-0.274	0.086	-0.021	0.110	-0.111	-0.039	1.000						
ALT/SGPT	0.050	0.074	.175^**^	0.091	-0.195	.180^*^	-0.080	-0.151	0.027	0.029	.134^*^	1.000					
INR	0.065	.249^**^	0.133	.231^**^	-0.198	-0.097	-0.108	0.163	0.065	0.055	0.141	0.003	1.000				
Platelets	0.053	0.004	-0.064	-0.086	0.234	-0.006	-0.008	0.005	0.052	-.172^**^	-.171^**^	-0.034	0.037	1.000			
Hematocrit	-0.089	0.035	.134^*^	-0.031	-0.201	0.151	-0.096	-0.187	0.053	-0.065	0.119	.287^**^	0.126	0.038	1.000		
WBC	.116^*^	.748^**^	.225^**^	.342^**^	0.171	.311^**^	.183^**^	0.043	-0.009	0.111	0.107	0.079	.243^**^	0.052	0.092	1.000	
Hb	-0.067	0.020	.175^**^	-0.080	-0.164	0.099	-.117^*^	-0.245	0.080	-.128^*^	.137^*^	.298^**^	0.080	-0.008	.855^**^	0.023	1.000

A single asterisk indicates that the correlation is significant at the 0.01 level (2-tailed). Double asterisks indicate that the correlation is significant at the 0.05 level (2-tailed).

### Chest CT Scan Findings

**Figure 2:**
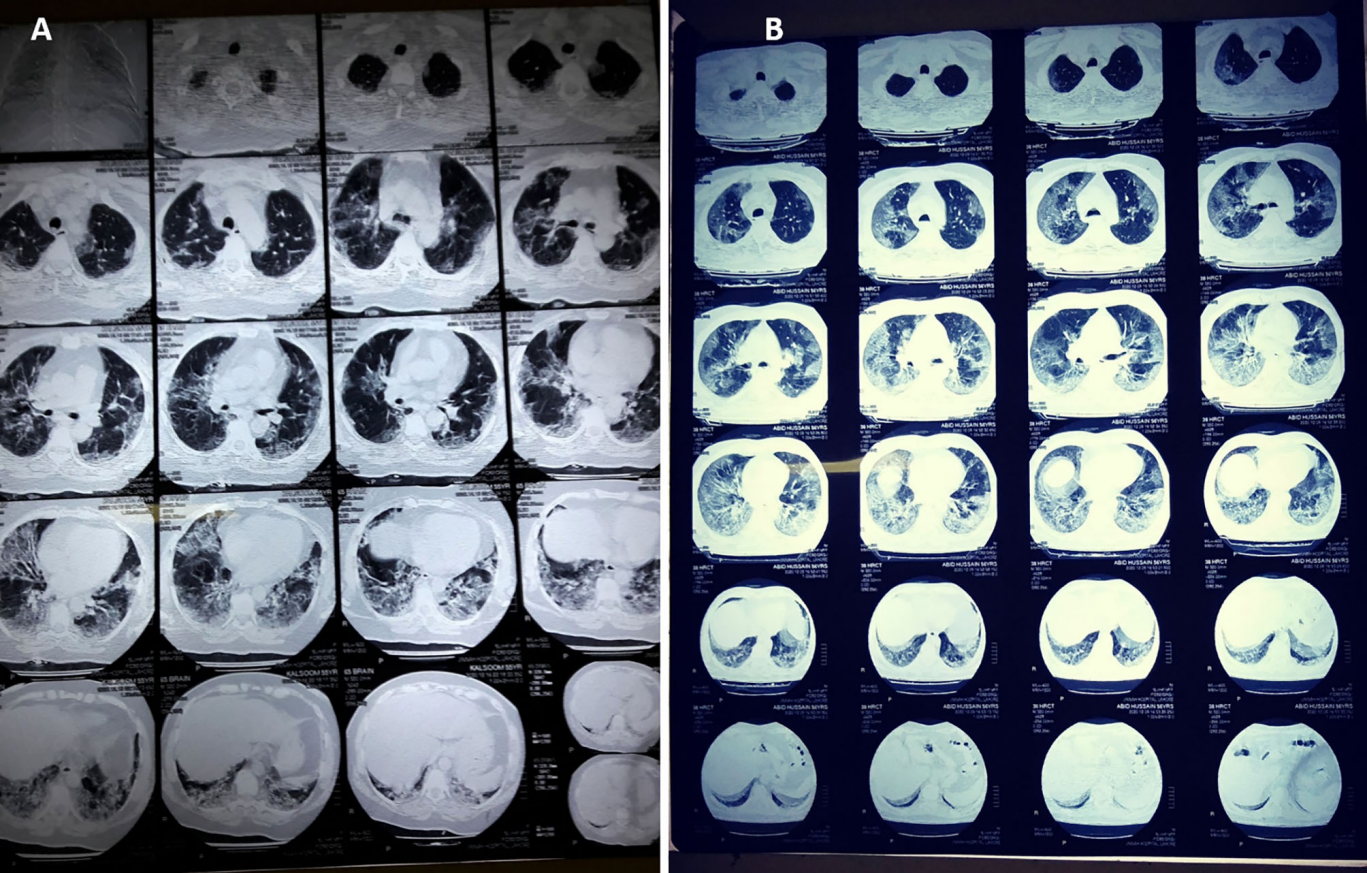
CT scan digital images of **(A)** mild-to-moderate and **(B)** severe SARS-CoV-2-infected patients. The percentage of lung involvement can be spotted significantly in both types of patient.

The patients who underwent chest CT scans were also assessed in this study, and the details are summarized in **Table 7** and **Figure 2**. The mean age of these patients was 57 ± 12.6 years, including 76.7% male patients and 23.3% females. The mean percentage of lung involvement in male patients was 51.3 ± 15.8), and it was 54.3 ± 14.3 in female patients. Only 20% of the patients had age ≤50 years (38.3 ± 7.7), while a high number of patients (80%) had age of >50 years (62.4 ± 8.1). The most common findings were ground glass opacity (GGO; *n* = 28, 93.3%), mixed pattern of consolidation (*n* = 12, 40%), and interlobular septal thickening (*n* = 6, 20%). A small percentage of patients had GGO and opacities (*n* = 2, 6.7%), infiltration (*n* = 2, 6.7%), consolidation (*n* = 1, 3.3%) and cavitation (*n* = 1, 3.3%). Seventeen (57.7%) had five affected lobes, 8 (26.7%) had four affected lobes, 2 (6.7%) had three affected lobes, 2 (6.7%) had two affected lobes, and 1 (3.3%) had one affected lobe **Figure 2**.

**Table 7:** Findings of the CT scan of 30 COVID-19 patients.

Findings	Number of Patients*n* = 30	Age		Gender		Severity	
		≤50 (*n* = 6)	>50 (*n* = 24)	Male (*n* = 23)	Female (*n* = 7)	Mild/moderate (*n* = 7)	Severe (*n* = 23)
Consolidation	1 (3.3)		1 (4.2)	1 (4.3)			1 (4.3)
Cavities	1 (3.3)		1 (4.2)	1 (4.3)			1 (4.3)
GGO and consolidation	12 (40)	3 (50)	9 (37.5)	10 (43.5)	2 (28.5)	2 (28.5)	10 (43.5)
GGO and opacities	2 (6.7)		2 (8.3)	1 (4.3)	1 (14.2)		2 (8.7)
Infiltration	2 (6.7)		2 (8.3)	2 (8.7)			2 (8.7)
GGO	28 (93.3)	6 (100)	22 (91.7)	21 (91.3)	7 (100)	6 (85.7)	22 (95.6)
GGO and nodule	1 (3.3)	1 (16.7)		1 (4.3)			1 (4.3)
Interlobular septal thickening	6 (20)	3 (50)	3 (12.5)	4 (17.4)	2 (28.5)		6 (26.1)
Lobes involved (0–5), *n* = 30	30 (100)	6 (100)	24 (100)	23 (100)	7 (100)	7 (100)	23 (100)
One lobe	1 (3.3)		1 (4.7)	1 (4.3)			1 (4.3)
Two lobes	2 (6.7)		2 (8.3)	2 (8.7)			2 (8.7)
Three lobes	2 (6.7)		2 (8.3)	2 (8.7)		1 (14.3)	1 (4.3)
Four lobes	8 (26.7)	1 (16.7)	7 (29.2)	8 (34.8)		3 (42.8)	5 (21.7)
Five lobes	17 (57.7)	5 (83.3)	12 (50)	10 (43.5)	7 (100)	3 (42.8)	14 (60.8)
Percentage of lung involvement, *n* (%) Mean (SD)	30 (100)	6 (100), 42.5 (17.2)	24 (100), 54.4 (14.1)	23(100), 51.3 (15.8)	7(100), 54.3(14.3)	7 (100), 44.3 (12.7)	23 (100), 54.3 (15.4)

Data are presented as n (%) and mean (SD).

## Discussion

### Demography

The number of male patients was high compared with that of female patients, presumably due to men’s increased vulnerability to COVID 19 due to various reasons (Gargaglioni and Marques, 2020; Pradhan and Olsson, 2020). It has been attributed that female patients showed more resistance to COVID-19 infection than men due to differences in sex hormones and a lower expression of receptors (ACE-2) (Bwire, 2020). Others have reported that men show high mortality from heart disease and diabetes, contributing to sex-based severity from COVID-19 (Pradhan and Olsson, 2020; Tian et al., 2020). The older-age peoples are at a high risk to catch SARS-CoV-2 due to their weak immune system and a high prevalence of comorbidities, and this susceptibility has also been concluded in different studies (Al Mutair et al., 2020; Mueller et al., 2020; Yegorov et al., 2021). A high number of COVID-19 patients in the study belonged to the extended family type, presumably due to crowded living conditions that favored the spread of disease. The same has been concluded in different studies (Noreen et al., 2020; Ramadhana, 2020).

The source of infection was unknown for 60% of the COVID-19 cases. It has been reported in other studies that the prevalence of asymptomatic carriers is difficult to determine and requires comprehensive screening. This would provide essential information on hidden viral strains circulating in the community, and the rate of such carriers would be different in high-density urban areas (Ahmed et al., 2020; Cloutier et al., 2020). Similarly, it has also been reviewed that the majority of asymptomatic patients appear to have a milder clinical course during hospitalization, but the severity of the symptoms in asymptomatic patients among all confirmed cases varies widely (from 1.95 to 87.9%), according to the study setting and the populations studied (Han et al., 2020). It was concluded that patients with a travel history from abroad (11%) were positive for COVID-19 on the health authorities’ investigation. Since Pakistan has a high frequency of travel and trade with China and Iran, it has been reported that the risk of viral transmission across borders increases (Noreen et al., 2020; Javed et al., 2021).

Furthermore, an inclination to withhold information regarding travel history to high-risk epidemic regions also results in unpredictable outcomes (Wei et al., 2020). The susceptibility of getting infected while coming into contact with confirmed COVID-19 patients (27%) may be attributed to person-to-person transmission, as reported in several similar studies (Cloutier et al., 2020; Lechien et al., 2020; Martinez-Fierro et al., 2021). It was concluded that the average number of days spent in the hospital was significantly high among severe patients compared with mild/moderate patients (10 *vs*. 5 days, *p* < 0.001).

### Clinical Features

Symptoms of illness, such as fever (80%), shortness of breath (75%), and cough (57%), are most common in patients, predominantly due to the reason that the disease is reported to affect the lower respiratory system (Petersen et al., 2020). Presumably, due to similar reasons, the symptoms of shortness of breath, pneumonia, and abnormal chest X-ray were significantly highly occurring in severe patients. The results coincide with those of other studies wherein it has been reported that fever, cough, fatigue, and shortness of breath are common symptoms in severe COVID-19 patients (Al Mutair et al., 2020; Durrani et al., 2020). The association of the lower respiratory system in affecting the severity of COVID-19 patients has also been reported in different studies (Marinari et al., 2019; Brosnahan et al., 2020; Hannawi et al., 2021).

Other symptoms of COVID-19 patients, including myalgia and generalized body aches, vomiting, headaches, *etc*., were presumably associated with symptoms of fever, cough, and shortness of breath. Similar findings were reported in various studies from different regions (Sun et al., 2020b; Weng et al., 2021). An alternative diagnosis should also be considered to prevent weak opinions—mainly other infectious diseases like pneumonia of bacterial etiology, bacteremia, respiratory infections such as exacerbation of COPD, and cardiovascular disorders like acute heart failure. Systematic evaluation of heart function should be done in COVID-19 suspected patients (Castiello et al., 2022). Ruling out other differences with similar clinical features prevents extended hospital stay in isolation wards and shortage of beds required for critical COVID-19 patients, thereby reducing the burden on the healthcare system.

### Comorbidities

There was a predominance of patients who had a long-standing history of hypertension (48.8% *vs*. 35.9%), type 2 diabetes (38.5 *vs*. 31.4%), and ischemic heart disease (16.9 *vs*. 12.5%). In severe COVID-19 patients, these may be linked to multiple factors, among them as the significant ones were the aged patients, reduced systemic oxygenation intake due to pneumonia, concomitantly increased cardiac demand, and use of ACE inhibitors (Ejaz et al., 2020). The high percentage of hypertension and COPD in severe patients may also be linked to similar reasons, along with underlying poor lung reserves or the increased expression of ACE-2 receptors in small airways (Leung et al., 2020; Perrotta et al., 2020; Gómez Antúnez et al., 2021). Many studies found a high risk of infection in the cardiovascular, pulmonary, and renal patients (De Almeida-Pititto et al., 2020; Leung et al., 2020). Most importantly, studies revealed that the high mortality ratios in COVD-19 patients were associated with the cardiovascular and renal complications of diabetes and independently with glycemic control and body mass index (Holman et al., 2020; Richardson et al., 2020; Dyusupova et al., 2021).

### Laboratory Parameters

Complete blood count, coagulation profile, biochemical parameters, and inflammatory mediators predict the disease severity and outcomes (Angioni et al., 2020; Zeng et al., 2020). Values of WBC, platelets, neutrophil, total bilirubin, CRP, and ferritin were significantly high in severe patients compared with those in mild/moderate cases. The study signifies the usefulness of biomarkers in assessing the severity of COVID-19 infection in patients, which may help to improve the treatment of the disease. The results of this study are in line with contrasting findings in similar studies (Yang et al., 2020; Hannawi et al., 2021; Yegorov et al., 2021). The increase of CRP and ferritin in critically ill COVID-19 patients has been measured in various findings (Al Mutair et al., 2020; Wang et al., 2020; Hannawi et al., 2021). The same tendency was observed for WBC, platelets, neutrophils, and bilirubin (Yin et al., 2020; Taj et al., 2021; Yegorov et al., 2021). A significantly positive correlation was observed between the ferritin and laboratory parameters, including Hb, WBC, ALT/SGP, total bilirubin, CRP, and LDH, while these were negatively correlated with lymphocytes. Low D-dimer, serum creatinine, and LDH were observed in severe patients compared with mild-to-moderate patients. This may be because the regular administration of anticoagulant drugs for treatment in severe patients decreased the levels of D-dimers in them presumably (Mouhat et al., 2020). Similar results were obtained in studies conducted to assess on-protocol COVID-19 patients (Kamel et al., 2021; Tassiopoulos et al., 2021). Apparently, due to similar reasons, the D-dimer had a highly significant positive correlation with WBC, INR, procalcitonin, and LDH. A retrospective study by Taj et al. (2021) on confirmed COVID-19 patients concluded that leukocytosis, neutrophilia, elevated neutrophil-to-lymphocyte ratio, activated partial thromboplastin time, D-dimer, lactate dehydrogenase, serum ferritin, and CRP are associated with the severity of the disease.

Procalcitonin (PCT) is a food and drug administration (FDA)-approved diagnostic marker for different diseases. As per FDA executive summary (Gaithersburg, 2016), the normal range of PCT is <0.05 to <0.1 ng/ml. Therefore, any elevation of PCT beyond <0.1 ng/ml as shown in the current study (mild/moderate = 0.21 ng/ml, severe/critical = 2.79 ng/ml, and normal range = 0.1 ng/ml) should be taken as clinically significant because it is linked with disease severity.

### Medications Used in the Treatment

A significantly high percentage of severe patients were treated with steroid (dexamethasone), antiviral (remdesivir), antibiotic (meropenem), anticoagulant (heparin), and immunosuppressive (tocilizumab) compared with mild/moderate patients. Dexamethasone helps control the inflammation of the lower respiratory tract with its immunosuppressive role (Selvaraj et al., 2020). Similarly, the use of remdesivir had provided evidence of lowering the respiratory tract infection (Beigel et al., 2020). The controlled use of antibiotics is reported to have safely and effectively treated most bacterial cases of pneumonia. Antibiotics have played an effective role in treating superimposed bacterial infections, such as bacterial cases of pneumonia in COVID-19 patient settings (Beovic et al., 2020; Ginsburg and Klugman, 2020). Activation of the coagulation cascade leading to severe hypercoagulability has been detected in COVID-19 patients (Kichloo et al., 2020). Therefore, it has been recommended that early anticoagulation may reduce coagulopathy, micro-thrombus formation, and the risk of organ damages (Becker, 2020; Gozzo et al., 2020; Richardson et al., 2020). It has been reported that treatment with tocilizumab, whether administered intravenously or subcutaneously, might reduce the risk of invasive mechanical ventilation or death in patients with severe COVID-19 pneumonia (Guaraldi et al., 2020).

### Radiological Findings

#### Chest X-ray

Chest X-ray is a valuable tool in assessing disease progression and severity (Borghesi and Maroldi, 2020). In COVID-19 patients, it may be normal initially but may later follow a characteristic pattern of progression from the bilateral lower predominant zones to the upper zones, vertically extending peripheral to diffuse in critical ICU patients, thus showing a picture of ARDS (Durrani et al., 2020). It has been concluded that, in the initial chest X-ray on admission, the involvement of >4 zones has been linked to increased severity and an unfavorable outcome.

#### Chest CT Scan

Non-enhanced chest CT is a vital component in the diagnosis of patients suspected of a COVID-19 infection (Korevaar et al., 2020; Pruis et al., 2020). The pattern of GGO, GGO, and consolidation and interlobular septal thickening was the most prominent finding among the infected patients of different age groups, gender, and severity. The pattern of the findings is somewhat similar to that described in related studies on severe acute respiratory syndrome (Bernheim et al., 2020; Pan et al., 2020; Wu et al., 2020). In critical patients, a honey-combing pattern, traction bronchiectasis, and interlobar pleural traction can be observed. A follow-up CT after 6 months may show fibrotic changes in such patients (Ginsburg and Klugman, 2020; Han et al., 2021). A combination RT-PCR analysis and chest CT scan increases the sensitivity and specificity of COVID-19 diagnosis to 88 and 100%, respectively (He et al., 2020b). The semi-quantitative CT Severity Score System helps in showing the extent of pulmonary involvement. These are as follows:

0 score = no involvement

1 score = <5% involvement

2 score = 5–25% involvement

3 score = 26–49% involvement

4 score = 50–75% involvement

5 score = >75% involvement

The total CT severity score is calculated by summing up the individual lobe score, with the cumulative score ranging from 0 to 25. A CT score >18 has been correlated with increased severity, mortality, and worse prognosis (Saeed et al., 2021). In another study, it has been reported that a CT score >7 has been linked to increased chances of developing the post-COVID syndrome (Ali and Ghonimy, 2021).

## Conclusion

Older patients, predominantly male, were more in number in both groups with comorbidities, mainly hypertension, type 2 diabetes, and ischemic heart. The preventive measures against COVID-19 must be followed, and surveillance for asymptomatic carriers should be increased. The excessive use of antibacterial, antiviral, anticoagulant, and drugs for pain management was noted. Early detection based on laboratory-based biomarkers may prevent the severity of the illness.

## Data Availability Statement

The original contributions presented in the study are included in the article/**Supplementary Material**. Further inquiries can be directed to the corresponding author.

## Ethics Statement

This project was conducted under approval number UHS/REG-20/ERC/1758 from the University of Health Sciences Lahore Ethical Review Committee. The patients/participants provided their written informed consent to participate in this study.

## Author Contributions

All authors contributed to the article and approved the submitted version.

## Funding

This study is part of the Rapid COVID Project (RRG# 211) funded by the Higher Education Commission and the World Bank. The funder was not involved in the study design, collection, analysis, and interpretation of data, the writing of this article or the decision to submit it for publication.

## Conflict of Interest

The authors declare that the research was conducted in the absence of any commercial or financial relationships that could be construed as a potential conflict of interest.

## Publisher’s Note

All claims expressed in this article are solely those of the authors and do not necessarily represent those of their affiliated organizations, or those of the publisher, the editors and the reviewers. Any product that may be evaluated in this article, or claim that may be made by its manufacturer, is not guaranteed or endorsed by the publisher.
