# Supplementary material for: Epidemiology, Clinico-Pathological Characteristics, and Comorbidities of SARS-CoV-2-Infected Pakistani Patients
Source: Front Cell Infect Microbiol. 2022 May 26;12:800511. doi: 10.3389/fcimb.2022.800511 (PMC9226825; doi:10.3389/fcimb.2022.800511)
Supplement: Supplementary file 1 [file DataSheet_1.docx]

**Frequency of Co-morbidities in study population**

| **Co-morbidities** | **Frequency**  **n%** |
| --- | --- |
| IHD | 66 (14) |
| Immunocompromised state | 10(2.2) |
| Type 2 Diabetes Mellitus | 157 (34%) |
| Liver disease | 3(0.7) |
| COPD | 8(1.8) |
| Asthma (mod to severe) | 11(2.4) |
| smoking | 5(1.1) |
| HTN | 190(42) |

**Comparison of laboratory parameters in Covid19 patients with and without HTN**

|  | HTN | n | Mean | Std. Deviation | Std. Error Mean | p value |
| --- | --- | --- | --- | --- | --- | --- |
| Creatinine | Yes | 142 | 1.2508 | 1.20818 | .10139 | .304 |
|  | No | 205 | 1.1256 | 1.04451 | .07295 |  |
| Hb g/dl | Yes | 163 | 12.3107 | 2.36512 | .18525 | **.008** |
|  | No | 219 | 12.9174 | 2.05573 | .13891 |  |
| WBC/cubic mm | Yes | 163 | 13101.9080 | 6966.74251 | 545.67739 | .087 |
|  | No | 218 | 11831.7982 | 7298.29377 | 494.30283 |  |
| Haematocrit % | Yes | 148 | 37.1453 | 6.87787 | .56536 | .130 |
|  | No | 202 | 38.2010 | 6.08129 | .42788 |  |
| Platelets/mcL AD | Yes | 153 | 253903.9216 | 98589.63587 | 7970.49965 | .841 |
|  | No | 208 | 255996.6442 | 97788.60330 | 6780.41968 |  |
| APTT/Sec | Yes | 68 | 32.46 | 12.753 | 1.546 | .569 |
|  | No | 90 | 31.43 | 9.895 | 1.043 |  |
| PT/Sec | Yes | 71 | 15.8437 | 9.13254 | 1.08383 | .644 |
|  | No | 94 | 15.1945 | 8.74141 | .90161 |  |
| INR | Yes | 59 | 1.2575 | .49879 | .06494 | .425 |
|  | No | 87 | 2.7113 | 13.92174 | 1.49257 |  |
| ALT/SGPT(U/L0) | Yes | 135 | 38.8741 | 25.48615 | 2.19350 | **.003** |
|  | No | 190 | 49.6000 | 34.96753 | 2.53681 |  |
| T. bilirubin | Yes | 136 | .6729 | .82413 | .07067 | .558 |
|  | No | 188 | .6291 | .52078 | .03798 |  |
| Urea-N mg/dl | Yes | 71 | 26.8849 | 17.35926 | 2.06017 | .323 |
|  | No | 111 | 24.3207 | 16.79282 | 1.59390 |  |
| Lactate | Yes | 7 | 31.60 | 20.523 | 7.757 | .875 |
|  | No | 4 | 33.60 | 17.691 | 8.845 |  |
| Urea | Yes | 75 | 55.73 | 41.511 | 4.793 | .074 |
|  | No | 109 | 46.44 | 28.633 | 2.743 |  |
| Sodium | Yes | 138 | 139.4043 | 9.32475 | .79378 | .505 |
|  | No | 184 | 138.7918 | 7.15378 | .52738 |  |
| Potassium | Yes | 136 | 4.5032 | .93289 | .07999 | .795 |
|  | No | 180 | 4.4769 | .85924 | .06404 |  |
| Procalcitonin Adm | Yes | 23 | 8.18 | 31.201 | 6.506 | .171 |
|  | No | 41 | 1.36 | 4.175 | .652 |  |
| CRP | Yes | 151 | 95.7105 | 106.79517 | 8.69087 | .141 |
|  | No | 201 | 81.6770 | 71.31412 | 5.03011 |  |
| LDH | Yes | 78 | 577.40 | 449.569 | 50.904 | .964 |
|  | No | 82 | 574.78 | 273.875 | 30.244 |  |
| Creatine Kinase Adm | Yes | 1 | 1.20 | . | . | NA |
|  | No | 0^a^ | . | . | . |  |
| Troponin | Yes | 20 | 50.72 | 118.741 | 26.551 | .727 |
|  | No | 28 | 66.99 | 180.897 | 34.186 |  |
| ESR | Yes | 8 | 48.13 | 30.717 | 10.860 | .919 |
|  | No | 8 | 46.63 | 27.344 | 9.668 |  |
| D-Dimer | Yes | 153 | 1278.9442 | 1827.06580 | 147.70952 | .370 |
|  | No | 211 | 1492.9943 | 2505.69397 | 172.49909 |  |
| Ferritin Adm | Yes | 146 | 1040.9534 | 1074.97880 | 88.96588 | .923 |
|  | No | 209 | 1053.6900 | 1307.34807 | 90.43116 |  |
| a. t cannot be computed because at least one of the groups is empty. | | | | | |  |

**Comparison of laboratory parameters in Covid19 patients with and without Type 2 Diabetes Mellitus**

|  | Type 2 Diabetes Mellitus | N | Mean | Std. Deviation | Std. Error Mean | P value |
| --- | --- | --- | --- | --- | --- | --- |
| Creatinine | Yes | 115 | 1.1941 | 1.00066 | .09331 | .839 |
|  | No | 232 | 1.1683 | 1.16867 | .07673 |  |
| Hb g/dl | Yes | 134 | 12.6906 | 2.31724 | .20018 | .835 |
|  | No | 248 | 12.6412 | 2.15538 | .13687 |  |
| WBC/cubic mm | Yes | 132 | 13021.4394 | 8971.58393 | 780.87615 | .201 |
|  | No | 249 | 12032.5823 | 6005.12982 | 380.55964 |  |
| Haematocrit % | Yes | 124 | 38.2090 | 6.18479 | .55541 | .329 |
|  | No | 226 | 37.5053 | 6.57871 | .43761 |  |
| Platelets/mcL AD | Yes | 123 | 257801.6260 | 99602.70009 | 8980.87302 | .708 |
|  | No | 238 | 253718.4958 | 97340.08250 | 6309.62039 |  |
| APTT/Sec | Yes | 48 | 34.02 | 14.914 | 2.153 | .111 |
|  | No | 110 | 30.94 | 9.025 | .861 |  |
| PT/Sec | Yes | 48 | 17.8396 | 13.98969 | 2.01924 | **.028** |
|  | No | 117 | 14.5032 | 5.40140 | .49936 |  |
| INR | Yes | 45 | 1.2802 | .61806 | .09213 | .529 |
|  | No | 101 | 2.4996 | 12.92041 | 1.28563 |  |
| ALT/SGPT(U/L0) | Yes | 105 | 42.3524 | 29.43539 | 2.87260 | .274 |
|  | No | 220 | 46.4773 | 32.81990 | 2.21272 |  |
| T. bilirubin | Yes | 105 | .5794 | .41540 | .04054 | .202 |
|  | No | 219 | .6801 | .75387 | .05094 |  |
| Urea-N mg/dl | Yes | 62 | 24.6940 | 15.83214 | 2.01068 | .722 |
|  | No | 120 | 25.6450 | 17.65011 | 1.61123 |  |
| Lactate | Yes | 8 | 28.72 | 20.258 | 7.162 | .319 |
|  | No | 3 | 41.97 | 10.496 | 6.060 |  |
| Urea | Yes | 62 | 53.89 | 39.589 | 5.028 | .309 |
|  | No | 122 | 48.37 | 31.888 | 2.887 |  |
| Sodium | Yes | 115 | 138.5078 | 10.07453 | .93945 | .370 |
|  | No | 207 | 139.3580 | 6.85607 | .47653 |  |
| Potassium | Yes | 114 | 4.4505 | .95084 | .08905 | .572 |
|  | No | 202 | 4.5096 | .85600 | .06023 |  |
| Procalcitonin Adm | Yes | 19 | 8.71 | 34.310 | 7.871 | .183 |
|  | No | 45 | 1.74 | 4.674 | .697 |  |
| CRP | Yes | 128 | 99.2765 | 107.16974 | 9.47256 | .063 |
|  | No | 224 | 81.0802 | 75.14688 | 5.02096 |  |
| LDH | Yes | 64 | 561.18 | 298.473 | 37.309 | .678 |
|  | No | 96 | 585.98 | 410.423 | 41.889 |  |
| Creatine Kinase Adm | Yes | 1 | 1.20 | . | . | NA |
|  | No | 0^a^ | . | . | . |  |
| Troponin | Yes | 15 | 56.02 | 136.509 | 35.246 | .902 |
|  | No | 33 | 62.11 | 167.075 | 29.084 |  |
| ESR | Yes | 4 | 43.50 | 31.649 | 15.825 | .762 |
|  | No | 12 | 48.67 | 28.224 | 8.148 |  |
| D-Dimer | Yes | 130 | 1533.7944 | 2352.31748 | 206.31189 | .408 |
|  | No | 234 | 1330.3718 | 2185.28460 | 142.85652 |  |
| Ferritin Adm | Yes | 119 | 967.9732 | 890.02761 | 81.58870 | .376 |
|  | No | 236 | 1089.0322 | 1350.24828 | 87.89368 |  |
| a. t cannot be computed because at least one of the groups is empty. | | | | | |  |

**Comparison of laboratory parameters in Covid19 patients with and without IHD**

|  | IHD | N | Mean | Std. Deviation | Std. Error Mean | p value |
| --- | --- | --- | --- | --- | --- | --- |
| Creatinine | Yes | 48 | 1.3208 | .66751 | .09635 | .336 |
|  | No | 299 | 1.1537 | 1.16952 | .06764 |  |
| Hb g/dl | Yes | 55 | 12.6236 | 2.25954 | .30468 | .900 |
|  | No | 327 | 12.6644 | 2.20573 | .12198 |  |
| WBC/cubic mm | Yes | 56 | 13078.6071 | 5864.13713 | 783.62829 | .428 |
|  | No | 325 | 12253.9723 | 7380.29987 | 409.38538 |  |
| Haematocrit % | Yes | 51 | 38.7843 | 6.61611 | .92644 | .217 |
|  | No | 299 | 37.5790 | 6.40640 | .37049 |  |
| Platelets/mcL AD | Yes | 51 | 249215.6863 | 102530.83706 | 14357.18891 | .644 |
|  | No | 310 | 256079.3613 | 97369.64572 | 5530.22426 |  |
| APTT/Sec | Yes | 25 | 33.72 | 7.994 | 1.599 | .369 |
|  | No | 133 | 31.53 | 11.683 | 1.013 |  |
| PT/Sec | Yes | 30 | 16.6633 | 9.25357 | 1.68946 | .419 |
|  | No | 135 | 15.2095 | 8.82098 | .75919 |  |
| INR | Yes | 28 | 1.3489 | .67445 | .12746 | .673 |
|  | No | 118 | 2.3076 | 11.95566 | 1.10061 |  |
| ALT/SGPT(U/L0) | Yes | 49 | 43.3673 | 22.30442 | 3.18635 | .672 |
|  | No | 276 | 45.4601 | 33.20200 | 1.99853 |  |
| T. bilirubin | Yes | 50 | .6044 | .32803 | .04639 | .619 |
|  | No | 274 | .6553 | .70886 | .04282 |  |
| Urea-N mg/dl | Yes | 24 | 35.6917 | 18.21514 | 3.71815 | **.001** |
|  | No | 158 | 23.7458 | 16.31579 | 1.29801 |  |
| Lactate | Yes | 2 | 26.36 | 15.330 | 10.840 | .642 |
|  | No | 9 | 33.66 | 19.856 | 6.619 |  |
| Urea | Yes | 19 | 71.00 | 34.866 | 7.999 | **.006** |
|  | No | 165 | 47.84 | 33.936 | 2.642 |  |
| Sodium | Yes | 45 | 140.7089 | 9.02738 | 1.34572 | .142 |
|  | No | 277 | 138.7856 | 7.98056 | .47951 |  |
| Potassium | Yes | 44 | 4.4886 | 1.14770 | .17302 | .998 |
|  | No | 272 | 4.4882 | .84403 | .05118 |  |
| Procalcitonin Adm | Yes | 10 | 15.53 | 47.209 | 14.929 | .033 |
|  | No | 54 | 1.64 | 4.581 | .623 |  |
| CRP | Yes | 49 | 91.7939 | 97.06914 | 13.86702 | .727 |
|  | No | 303 | 87.0345 | 87.11094 | 5.00439 |  |
| LDH | Yes | 23 | 575.00 | 240.545 | 50.157 | .988 |
|  | No | 137 | 576.23 | 386.971 | 33.061 |  |
| Creatine Kinase Adm | Yes | 0^a^ | . | . | . | NA |
|  | No | 1 | 1.20 | . | . |  |
| Troponin | Yes | 11 | 23.28 | 30.002 | 9.046 | .379 |
|  | No | 37 | 71.19 | 176.860 | 29.076 |  |
| ESR | Yes | 1 | 55.00 | . | . | .790  . |
|  | No | 15 | 46.87 | 29.014 | 7.491 |  |
| D-Dimer | Yes | 55 | 1664.7727 | 2715.45321 | 366.15164 | .349 |
|  | No | 309 | 1356.4329 | 2152.71859 | 122.46388 |  |
| Ferritin Adm | Yes | 46 | 1350.8546 | 1010.94057 | 149.05506 | .070 |
|  | No | 309 | 1003.4339 | 1238.26332 | 70.44234 |  |
| a. t cannot be computed because at least one of the groups is empty. | | | | | |  |
